# The main e-cigarette component vegetable glycerin enhances neutrophil migration and fibrosis in endotoxin-induced lung injury via p38 MAPK activation

**DOI:** 10.1186/s12931-022-02307-z

**Published:** 2023-01-10

**Authors:** Vincent Yi-Fong Su, Wei-Chih Chen, Wen-Kuang Yu, Huai-Hsuan Wu, Hao Chen, Kuang-Yao Yang

**Affiliations:** 1 Faculty of Medicine, School of Medicine, National Yang Ming Chiao Tung University, No. 155, Sec 2, Linong St, Taipei, 11221 Taiwan; 2 Department of Internal Medicine, Taipei City Hospital, Taipei City Government, Taipei, Taiwan; 3 Department of Chest Medicine, Taipei Veterans General Hospital, No. 201, Sec. 2, Shi-Pai Road, Taipei, 11217 Taiwan; 4 Institute of Emergency and Critical Care Medicine, School of Medicine, National Yang Ming Chiao Tung University, Taipei, Taiwan; 5 Cancer Progression Research Center, National Yang Ming Chiao Tung University, Taipei, Taiwan; 6 Department of Exercise and Health Sciences, College of Kinesiology, University of Taipei, Taipei, Taiwan

**Keywords:** e-cigarette, Chemotaxis, Fibrosis, p38 MAPK, Acute lung injury, Vegetable glycerin

## Abstract

We investigated the effects of vegetable glycerin (VG), a main e-cigarette constituent, on endotoxin-induced acute lung injury (ALI). Mice received intratracheal administration of 30% VG in phosphate buffered saline (PBS) vehicle or only PBS (control) for 4 days. On Day 5, mice received an intratracheal instillation of lipopolysaccharide (LPS) (LPS group and VG + LPS group) or PBS (VG group and control group). Lung histopathology, expression of chemokine receptors, and regulatory signaling were analyzed 24 h after the Day 5 treatment. VG significantly increased ALI-associated histopathological and fibrotic changes in both the VG group and LPS-induced ALI mice (VG + LPS group). Immunohistochemistry (IHC) and western blot analyses revealed that VG administration resulted in upregulation of neutrophil markers [lymphocyte antigen 6 complex locus G6D (Ly6G) and myeloperoxidase (MPO)] as well as upregulation of the expression of transforming growth factor-β (TGF-β), a central mediator of fibrogenesis, in the lungs of both VG and VG + LPS groups. VG enhanced the expression of adhesion molecules [very late antigen 4 (VLA-4) and vascular cell adhesion molecule 1 (VCAM-1)] and increased activation of p38 mitogen-activated protein kinase (p38 MAPK) to prompt neutrophil recruitment in the lungs of mice with ALI. Intraperitoneal administration of a p38 inhibitor attenuated these histopathological changes significantly as well as VG-induced upregulation in expression of Ly6G, MPO, VLA-4, VCAM-1, TGF-β, and collagen-1 in mice with ALI. In conclusion, VG enhances neutrophil chemotaxis and fibrosis and it amplifies the inflammatory response associated with LPS-induced ALI in the lungs via enhancement of p38 MAPK activity.

**Supplementary Information:**

The online version contains supplementary material available at 10.1186/s12931-022-02307-z.

## Introduction

E-cigarette/vaping product use-associated lung injury (EVALI), originally known as vaping-associated pulmonary illness, is an acute or subacute respiratory disease that can be critical and fatal. A cluster of patients with a mysterious lung disease, that is now recognized as EVALI, was first described in July 2019 in the USA [1]. As of February 18, 2020, the US Centers for Disease Control and Prevention (CDC) had documented 2,807 cases of EVALI, including 68 that were fatal [2], and they cited vaping as the main cause. The vaped substances in E-cigarettes contain many substances, including additives such as vegetable glycerin (VG) and propylene glycol (PG) as well as flavoring ingredients, nicotine, cannabinoids (e.g., tetrahydrocannabinol, cannabidiol), and vitamin E acetate. Upon heating, VG and PG generate vapor and act as a carrier for nicotine and flavorings.

The key risk factor for EVALI is the use of e-cigarettes or similar products. However, the pathogenesis of EVALI still remains unclear. Many different pathological findings related to EVALI have been reported, including acute lung injury (ALI), diffuse alveolar damage, diffuse alveolar hemorrhage, organizing pneumonia, acute eosinophilic pneumonia, lipoid pneumonia, and respiratory-bronchiolitis interstitial lung disease [3]. Although the precise pathologic findings of EVALI may be diverse, there is some consistent evidence that warrants attention. The CDC reported that people using cannabinoid-containing vaping products are at a higher risk of developing severe acute respiratory distress syndrome (ARDS) secondary to EVALI. Recent evidence has implicated vitamin E acetate in e-cigarettes as a driver of severe ARDS development in patients with EVALI [4]. Nevertheless, the focus on e-cigarette compounds as chemical instigators of the EVALI outbreak is reasonable. E-cigarette fluids have been shown to contain many toxic compounds: nicotine, volatile organic compounds, trace metal, bacterial endotoxins, and fungal glucans. Additional experimental studies may provide information on whether exposure to other e-cigarette components can directly cause lung injury [5]. VG is generally used for aerosol production when vaporized. However, the safety of VG use remains unclear in the context of increasing reports of pulmonary inflammation in many e-cigarette users. The investigation on the effects of vaping on lung biology is required to establish clear public policy guidance and regulation [6].

ALI and ARDS are life-threatening diseases in critically ill patients. ALI/ARDS comorbidity is characterized by neutrophil recruitment into the lungs, interstitial edema, endothelial injury, and epithelial injury. The most common cause of ALI/ARDS is severe sepsis and septic shock caused by bacterial infection. Lipopolysaccharide (LPS) is an endotoxin from Gram-negative bacilli that acts as a strong chemotactic component for neutrophils and thus is a strong trigger of the pathogenesis of sepsis and septic shock [7]. Neutrophils, the inflammatory cells that respond earliest to sepsis, are recruited following an inflammatory stimulus in sepsis-induced ALI. Our previous work [8–13] has demonstrated that an intratracheal injection of LPS promotes neutrophilic migration resulting in extensive ALI in mice. However, the role of VG in sepsis-induced ALI is not fully understood. In the present report, we describe our investigation into the effects of VG on neutrophil chemotaxis in a mouse model of endotoxin-induced ALI.

## Results

### Effects of VG on the histopathological features of LPS-induced ALI

**Fig. 1:**
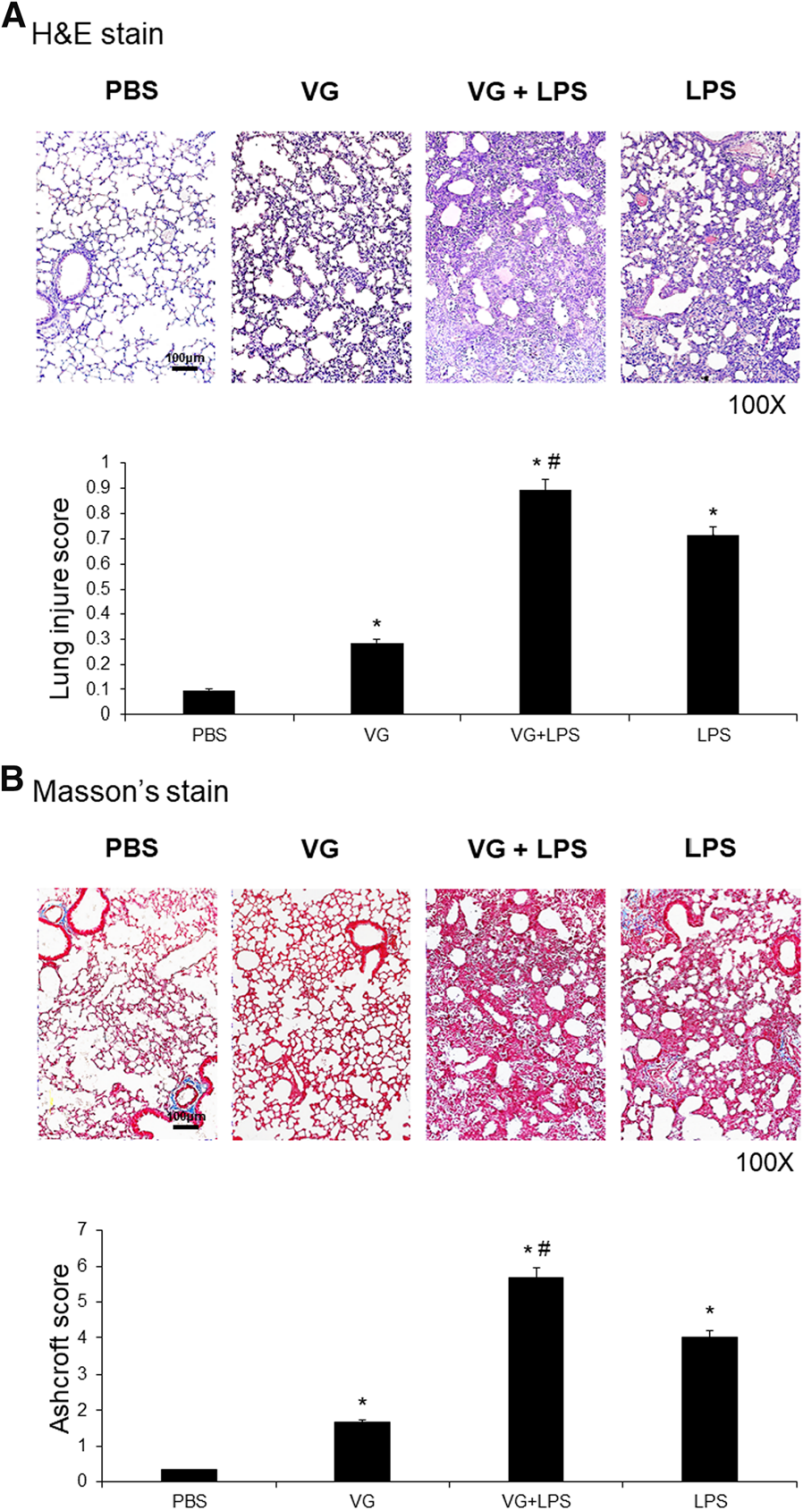
Histological examination of VG-induced lung injury. Administration of VG, a main e-cigarette component, enhanced the histological features in mice with LPS-induced ALI. **A** Histological examination of H&E-stained sections revealed that the lungs of mice injected with PBS (non-treated control group), VG (VG only group), LPS (LPS only group), and VG prior to LPS (VG + LPS group) had minimal histopathological abnormalities, mild ALI, pronounced ALI, and severe ALI, respectively, demonstrating that intratracheal VG amplified lung injury pathology in mice with LPS-induced ALI. **B** Ashcroft lung injury scoring of Masson's trichrome-stained sections likewise showed that VG both induced lung fibrosis in mice not exposed to LPS and exacerbated lung fibrosis in mice with LPS-induced ALI. Data sets are expressed as means ± standard deviations. *p < 0.05 vs. PBS, ^#^p < 0.05 vs. LPS; N = 6 per group

Examination of hematoxylin and eosin (H&E) stained lung sections from mice given an intratracheal LPS injection revealed ALI characterized by alveolar edema with neutrophil recruitment (Fig. 1A). Intratracheal administration of VG induced acute inflammatory response leading to mild ALI in mice. Significantly, mice that had received VG showed enhanced pathological changes in their lungs upon being subjected to LPS-induced ALI (Fig. 1A). Ashcroft scoring of Masson’s trichrome stained lung tissue samples revealed that substantial lung fibrosis was induced in all LPS-injected ALI mice. Mice given VG without an LPS injection had significantly increased lung fibrosis relative to vehicle controls injected with only phosphate buffered saline (PBS). Mice given VG prior to LPS injection exhibited dramatically more severe lung fibrosis than LPS-induced ALI mice not exposed to VG (Fig. 1B).

### Effects of VG on neutrophil accumulation and fibrogenesis in the lungs

**Fig. 2:**
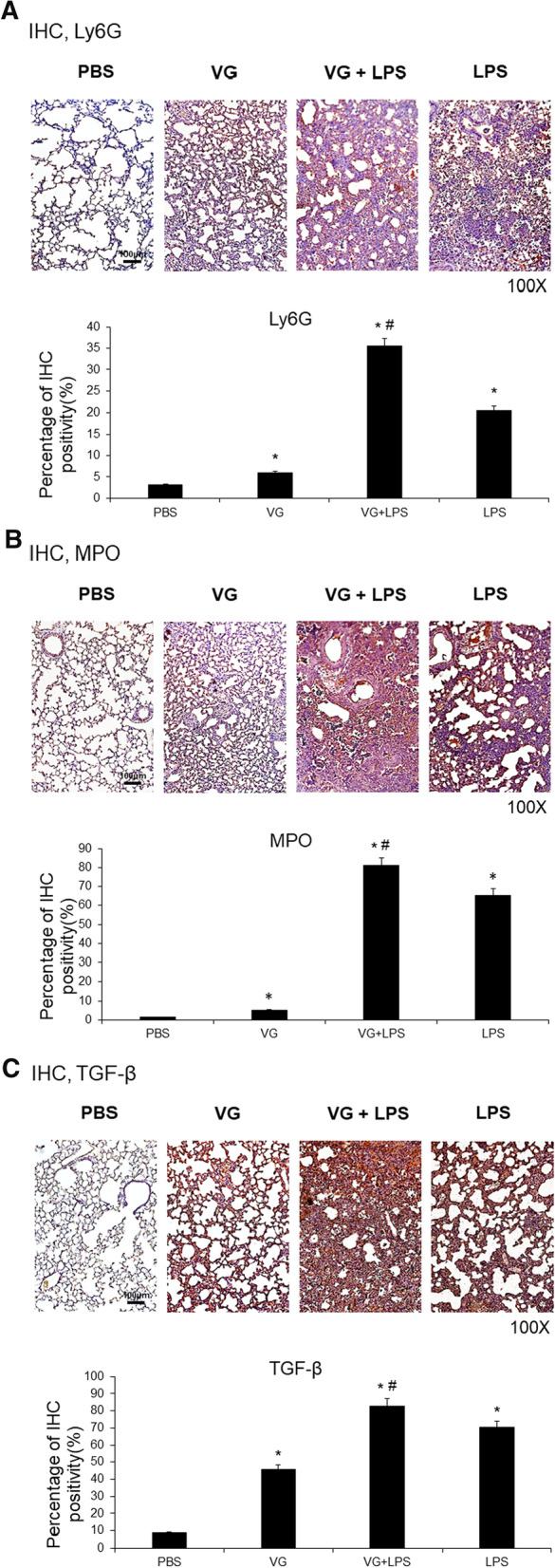
Neutrophil recruitment and fibrogenesis in VG-induced lung injury. VG administration increased expression of the neutrophil markers Ly6G and MPO and of the central mediator of fibrogenesis TGF-β in the lungs of mice with LPS-induced ALI. **A**–**C** Intratracheal injection of LPS increased expression of Ly6G, MPO, and TGF-β in the lungs. VG administration induced changes similar to those seen after LPS administration and enhanced the changes induced by LPS. Data sets are expressed as means ± standard deviations. *p < 0.05 vs. PBS, ^#^p < 0.05 vs. LPS; N = 6 per group

Immunohistochemistry (IHC) labelling of the common neutrophil markers lymphocyte antigen 6 complex locus G6D (Ly6G) and myeloperoxidase (MPO) (Fig. 2A and B) showed that, compared to control mice, VG-injected mice had slightly increased neutrophil recruitment in their lungs (Ly6G, 3.2% vs. 6.0%; MPO, 1.3% vs. 5.2%; both *p* < 0.05), while LPS-induced ALI mice had marked neutrophil accumulation in their lungs (Ly6G, 20.6%; MPO, 65.4%; both *p* < 0.05). Administration of VG augmented neutrophil accumulation in the lungs of LPS-induced ALI mice (VG + LPS: Ly6G, 35.5%; MPO, 81.2%; both *p* < 0.05).

IHC labelling of transforming growth factor-β (TGF-β), a central mediator of fibrogenesis, was performed to detect the induction of fibrotic changes in the lungs (Fig. 2C). Compared to control mice, markedly higher expression of TGF-β was found in the lungs of LPS-induced ALI mice (8.7% vs. 70.4%; *p* < 0.05) and mice treated with VG (TGF-β, 45.9%; *p* < 0.05). Prior administration of VG enhanced the expression of TGF-β in the lungs of LPS-induced ALI mice (VG + LPS: TGF-β, 82.9%; *p* < 0.05 vs. LPS).

### Effects of VG on neutrophil migration and fibrosis in the lungs

**Fig. 3:**
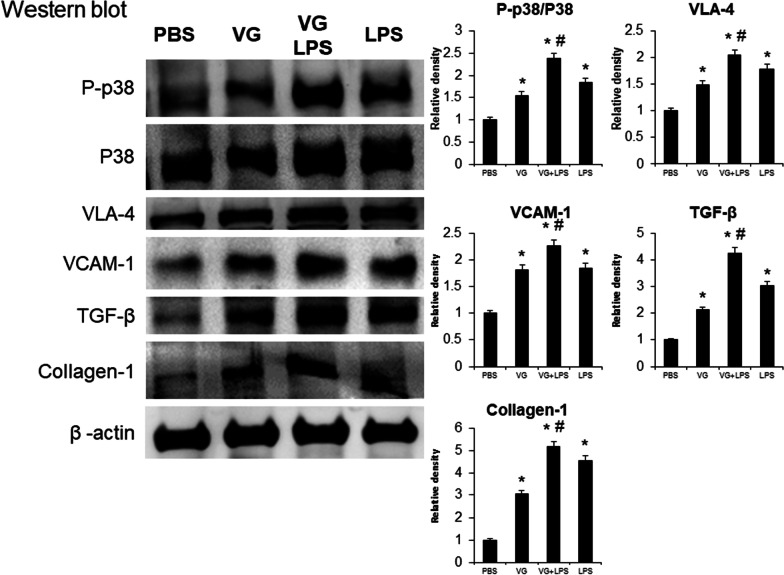
The activation of p38 MAPK in VG-induced lung injury. VG administration increased p-p38 MAPK activity and prompted neutrophil migration and fibrotic changes in mice with ALI. Western blots confirmed that the intratracheal injection of LPS increased the expression of adhesion molecules (VLA-4 and VCAM-1), and the central mediator of fibrogenesis (TGF-β), collagen-1, and the p-p38:p38 ratio, in the lungs of these mice. VG administration induced similar effects as LPS administration and enhanced the changes induced by LPS. *p < 0.05 vs. PBS, ^#^p < 0.05 vs. LPS; N = 6 per group

Western blot analysis of whole lung tissues showed that the expression levels of the adhesion molecules very late antigen 4 (VLA-4) and vascular cell adhesion molecule 1 (VCAM-1) were significantly increased 24 h after LPS-induced ALI induction compared with controls (both *p* < 0.05). Meanwhile, administration of VG increased the expression of these adhesion molecules significantly in both LPS untreated mice and in LPS treated mice (all *p* < 0.05). LPS- and VG-induced changes in the expression of fibrosis markers (TGF-β and collagen-1) paralleled the aforementioned adhesion molecule changes, including VG exacerbation of LPS-induced increases in fibrosis markers (all *p* < 0.05; Fig. 3).

### Pharmabological inhibition of inflammation and fibrogenesis in VG-induced lung injury

**Fig. 4:**
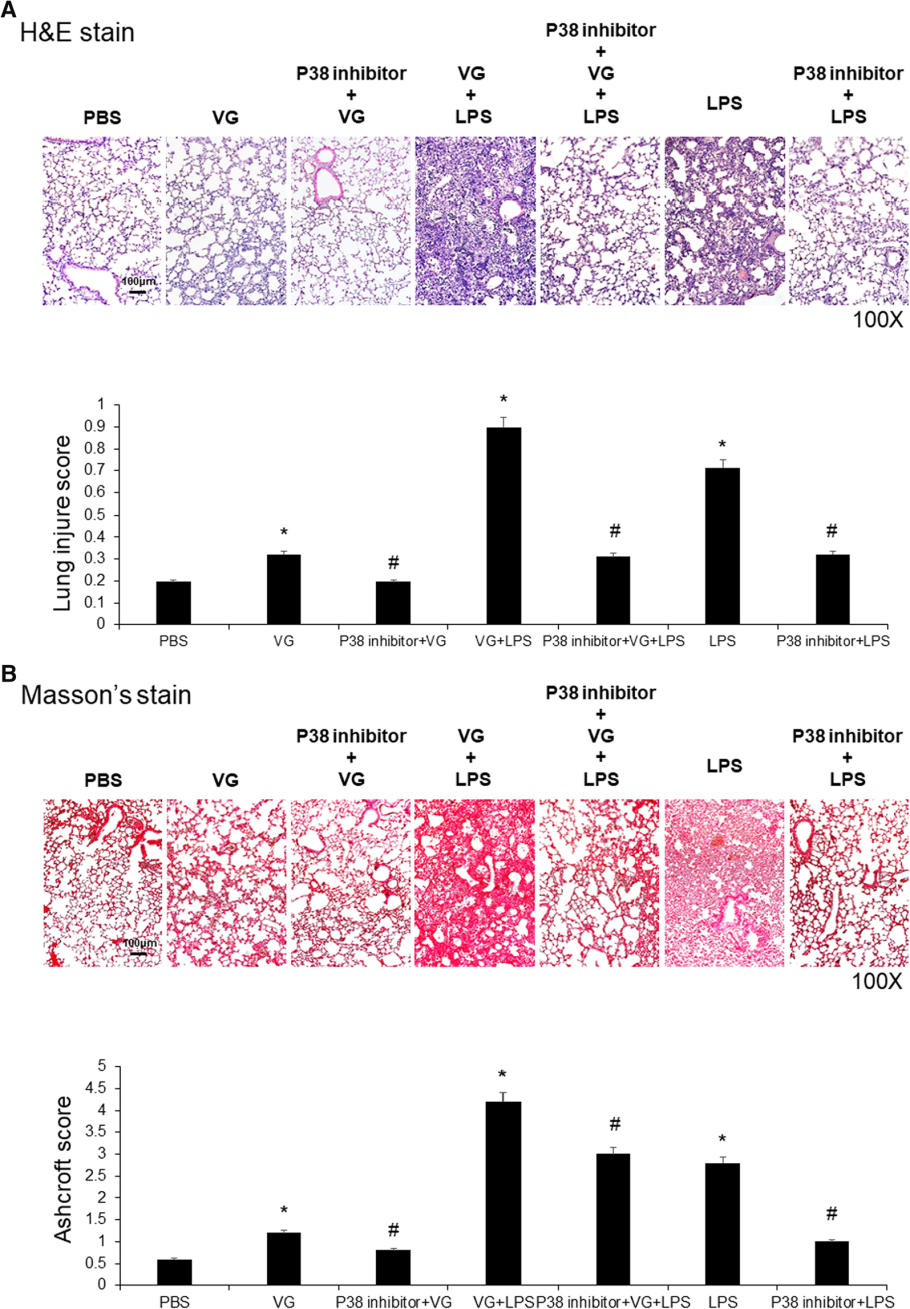
The inhibition of p38 MAPK activity attenuated histopathological changes in VG-induced lung injury. LPS and VG induced pathological changes of ALI and pulmonary fibrotic changes in mice. The administration of a p38 MAPK inhibitor restored these changes. **A** Examination of H&E stained sections showed that intratracheal administration of LPS or VG induced pathological changes. Intraperitoneal administration of a p38 MAPK inhibitor attenuated ALI changes. **B** Masson's trichrome staining revealed that intratracheal administration of LPS or VG induced fibrotic changes. Intraperitoneal administration of a p38 MAPK inhibitor attenuated lung fibrosis changes. *p < 0.05 vs. PBS, ^#^p < 0.05 vs. respective group without the inhibitor; N = 6 per group

Western blot analysis of whole lung tissues showed that activation of p38 mitogen-activated protein kinase (p38 MAPK), as determined by the ratio of phosphorylated p38 (p-p38) to total p38, was increased in mouse lungs following intratracheal administration of LPS or VG. The lungs of mice injected with both VG and LPS had greater p-p38:p38 levels than were found in the VG or LPS (only) groups (*p* < 0.05; Fig. 3). Intraperitoneal administration of a p38 inhibitor restored lung inflammation and lung injury severity, assessed in H&E-stained sections, in mice treated with LPS only, VG only, or LPS plus VG (all *p* < 0.05; Fig. 4A). Masson’s trichrome staining of the lung tissue confirmed that the administration of p38 inhibitor restored lung fibrosis severity grades in mice in all three lung-injuring treatment groups to levels that were significantly better than those seen in the respective groups not given the p38 inhibitor (all *p* < 0.05; Fig. 4B).

### Effects of p38 inhibitor on neutrophil migration and fibrosis in VG-induced lung injury

**Fig. 5:**
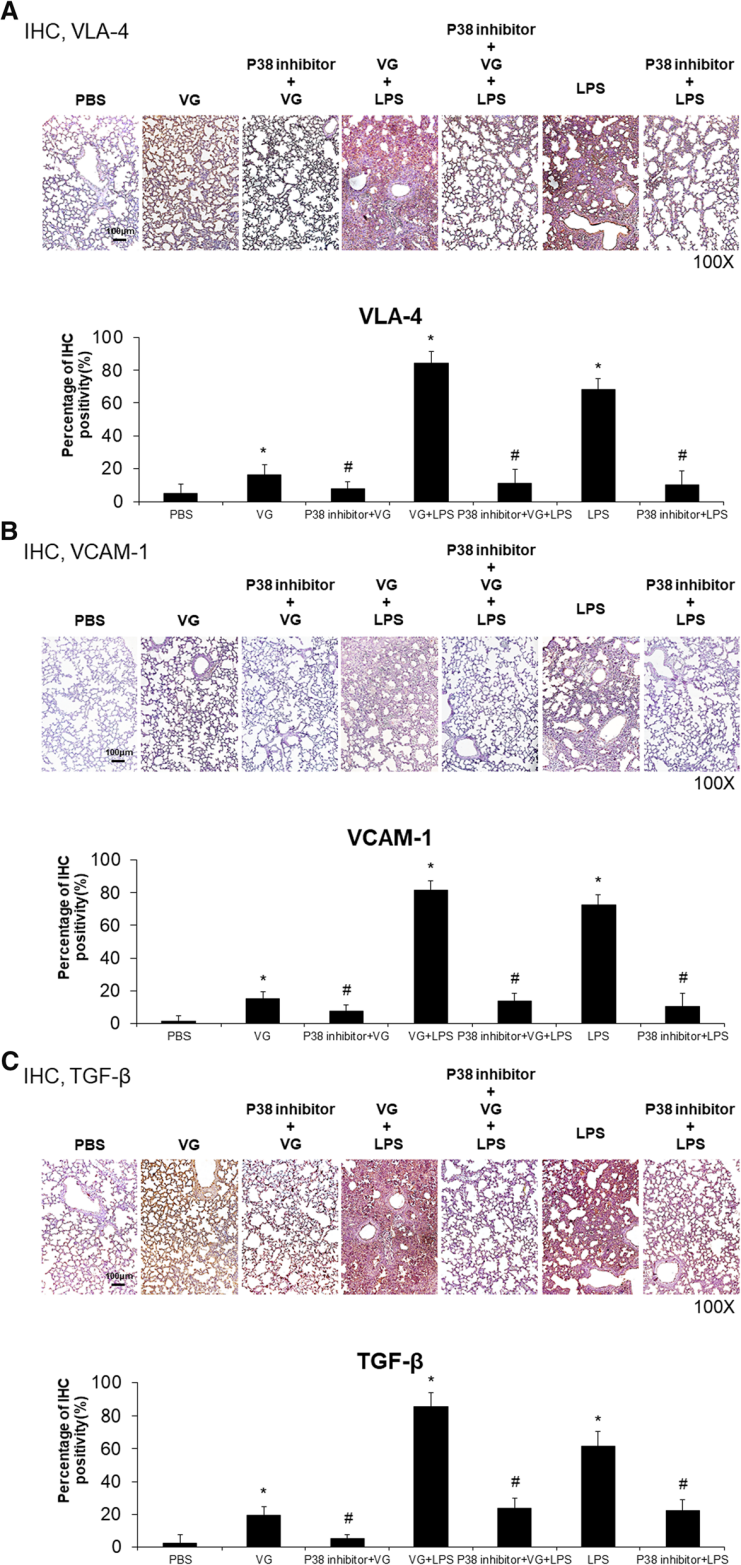
The inhibition of p38 MAPK activity attenuated neutrophil migration and fibrogenesis in VG-induced lung injury. Administration of a p38 MAPK inhibitor attenuated LPS and VG induced neutrophil migration and fibrogenesis. **A**–**C** Representative IHC images (above) and quantitative analysis summaries (below) showing LPS and VG induced increases in the expression of adhesion molecules VLA-4 (**A**) and VCAM-1 (**B**) and the expression of the central mediator of fibrogenesis TGF-β (**C**). Intraperitoneal administration of a p38 MAPK inhibitor countered these LPS and VG induced changes. *p < 0.05 vs. PBS, #p < 0.05 vs. respective group without the inhibitor; N = 6 per group

**Fig. 6:**
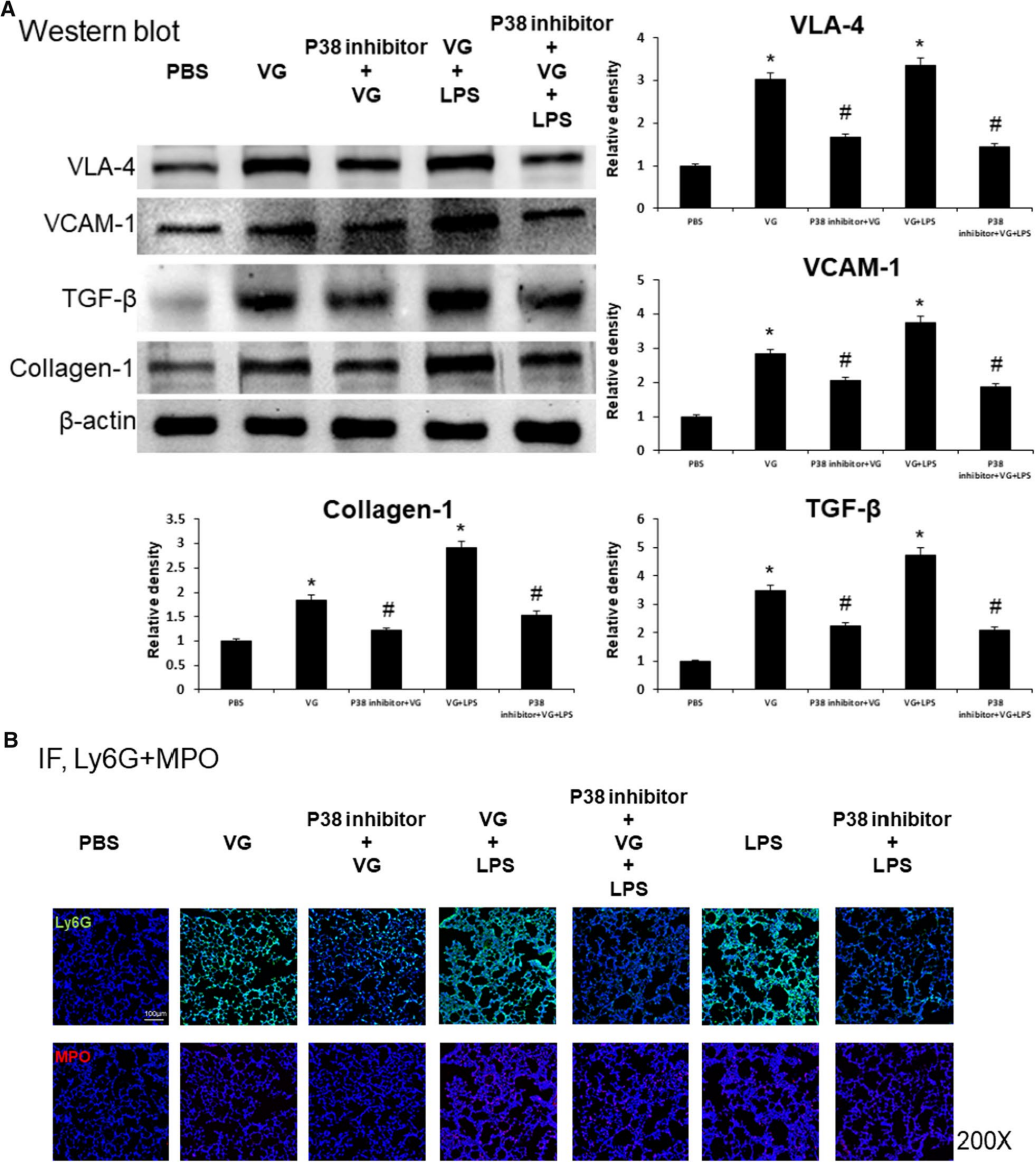
The inhibition of p38 MAPK activity attenuated neutrophil recruitment and fibrosis in VG-induced lung injury. Western blot and immunofluorescence analyses confirmed histology and IHC findings, including p38 inhibitor effects on LPS and VG induced neutrophil migration and fibrogenesis. **A** Western blot analyses showing that intraperitoneal administration of a p38 MAPK inhibitor attenuated the effects of intratracheal administration of LPS or VG on the expression of VLA-4 and VCAM-1 (adhesion molecules) as well as on the expression of the central mediator of fibrogenesis TGF-β and collagen-1 formation. **B** Immunofluorescence analyses showing that the p38 MAPK inhibitor inhibited LPS- and VG-induced increases in neutrophil recruitment (Ly6G and MPO). *p < 0.05 vs. PBS, ^#^p < 0.05 vs. respective group without the inhibitor; N = 6 per group

Analysis of IHC-labelled lung sections revealed that administration of a p38 MAPK inhibitor reduced neutrophil migration and fibrotic changes associated with lung injury damage induced by VG alone, LPS alone, or VG plus LPS. A p38 MAPK inhibitor significantly reduced the expression of VLA-4 (VG, 16.2% vs. VG + p38 inhibitor 8.1%; LPS, 68.4% vs. LPS + p38 inhibitor, 10.3%; VG + LPS, 84.3% vs. VG + LPS + p38 inhibitor, 11.3%; all *p* < 0.05; Fig. 5A), VCAM-1 (VG, 15.2% vs. VG + p38 inhibitor 7.3%; LPS, 72.4% vs. LPS + p38 inhibitor, 10.3%; VG + LPS, 81.3% vs. VG + LPS + p38 inhibitor, 13.5%; all *p* < 0.05; Fig. 5B), and TGF-β (VG, 19.3% vs. VG + p38 inhibitor 5.3%; LPS, 61.4% vs. LPS + p38 inhibitor, 22.3%; VG + LPS, 85.3% vs. VG + LPS + p38 inhibitor, 23.4%; all *p* < 0.05; Fig. 5C). Western blot analysis confirmed that VG induced increases in VLA-4, VCAM-1, and TGF-β expression and that p38 MAPK inhibition countered these changes in the lung tissue of mice with ALI (Fig. 6A). Immunofluorescence analysis showed that the p38 MAPK inhibitor down-regulated Ly6G and MPO expression in the lungs of mice with lung injury induced by VG alone, LPS alone, or VG plus LPS (Fig. 6B).

## Discussion

To the best of our knowledge, this study is the first to demonstrate that VG, the main ingredient in vaping substrates, enhances neutrophil migration in an ALI setting. This study offers four major contributions to the literature by showing that: (i) administration of VG induces pulmonary neutrophil recruitment in association with increased pathological severity of lung injury in LPS-induced ALI (Figs. 1, 2); (ii) VG increases the percentage of fibrotic changes in the lungs of mice with ALI (Figs. 1, 2); and (iii) VG upregulates VLA-4, VCAM-1, and collagen-1 expression levels in the lungs (Figs. 3, 4, 5) while (iv) it down-regulates p38 MAPK activity and upregulates TGF-β expression to induce neutrophil migration and fibrosis in mice with ALI (Figs. 4, 5,6).

Pathology findings described in EVALI cases include pneumonia, ALI, diffuse alveolar damage, hypersensitivity pneumonitis, eosinophilic pneumonia, lipoid pneumonia, respiratory-bronchiolitis interstitial lung disease, organizing pneumonia, and more [3, 14], suggesting that more than one lung injury mechanism may be involved. While the key risk factor for EVALI is e-cigarette use, the pathogenesis of EVALI remains unclear. The results of this study demonstrate that VG administration induces lung inflammation and fibrosis and increases ALI severity. Pulmonary neutrophil recruitment and fibrosis were predominant in VG-induced ALI. In a national US study [15] describing the pathological characteristics observed in 60 patients who died of EVALI, more than 70% of the patients had a white cell count > 11,000/mm^3^ and more than 60% had > 80% neutrophils. Thus, neutrophil chemotaxis should be considered an important pathway to EVALI. In our previous work [8–13], neutrophil migration into the lungs has been shown to play a critical role in the acute inflammatory response of ALI. It is evident that p38 MAPK enhances neutrophil chemotaxis by activating the expression adhesion molecules [16].

VG added in recommended amounts to food is considered safe. Because of the delicate nature of the lungs, even VG may be damaging [17]. ADDIN EN.CITE [18]. In a preclinical study [19], VG induced increased mucin production and airway remodeling as well as goblet cell metaplasia/hyperplasia in human lung. E-cigarette vapour extract increases neutrophil CD11b and CD66b expression, and causes a pro-inflammatory response from human neutrophils [20]. This study demonstrates that p38 MAPK activated in response to VG administration triggers neutrophil chemotaxis and inflames lung tissue via activation of VLA-4 and VCAM-1. Adhesion molecules, including VLA-4 and VCAM-1, play a major role in neutrophil migration, but such migration into the lungs can be suppressed by inhibiting the expression of adhesion molecules in an ALI setting [13]. Conversely, p38 MAPK induces neutrophil migration via upregulation of VLA-4 and VCAM-1 expression.

This study also demonstrates that VG exacerbates the severity of LPS-induced pulmonary fibrosis and collagen-1 accumulation. The present report provides the first evidence, to our knowledge, of VG enhancement neutrophil migration and fibrosis in LPS-induced ALI. Previously, we found that stem cell therapy inhibited neutrophil migration by down-regulating p38 MAPK activity in circulating neutrophils in mice with LPS-induced ALI [8], and that anti-fibrotic drugs could reduce LPS-induced pulmonary fibrosis severity by down-regulating p38 MAPK activity [13]. In contrast, VG enhanced neutrophil migration and collagen accumulation in the lungs of LPS-induced ALI mice, in part by enhancing p38 MAPK activity. Our findings suggest that VG has a specific pro-inflammatory effect that involves activation of the p38 MAPK pathway. These findings reflect the effects of VG on the development of EVALI. Therefore, we believe that p38 MAPK plays an additional role in promoting neutrophil recruitment in EVALI. However, administration of a p38 MAPK inhibitor partially reduced neutrophil migration and fibrotic changes induced by VG. VG-induced lung injury is mediated partly by the activation in p38 MAPK signaling pathway. VG-induced pulmonary inflammation and fibrosis is mild. However, the effects of VG can accumulate over time and lead to chronic lung diseases. Our future studies will attempt to identify additional mechanisms that induce the development of EVALI, and this in turn may prove to be important for therapeutic purposes.

This study has several limitations. EVALI pathophysiology is complex and involves various types of pathological findings. We explored VG effects on neutrophil chemotaxis; however, these results do not represent all effects of e-cigarettes on inflammation in the setting of endotoxin-induced ALI. Additional studies focusing on other e-cigarette constituents that may damage lung tissue are warranted.

## Materials and methods

### Experimental animals

Male C57BL/6 mice aged 8–12 weeks were purchased from the National Experimental Animal Center (Taipei, Taiwan) and housed at the Laboratory Animal Center of Taipei Veterans General Hospital (Taipei, Taiwan). They were kept under a 12/12-h light/dark cycle and had access to food and water ad libitum. All experimental procedures followed committee-approved protocols for institutional animal care and use (Taipei Veterans General Hospital IACUC no. 2020-258).

### Experimental design

Endotoxin-induced lung injury in mice is an experimental animal model of ALI. Here, we used a model of endotoxin-induced ALI established in our previous work [8–13]. Briefly, to induce ALI, anesthetized mice received an intratracheal instillation of LPS from *Escherichia coli* (0111:B4; Sigma-Aldrich, St. Louis, MO) at a dose of 5 mg/kg in 50 µL PBS. Control mice received intratracheal instillations of 50 µL PBS daily for 5 days. In the LPS group, mice received 50 µL PBS daily for four days (Days 1–4) and were treated with ALI-inducing LPS on Day 5.

E-cigarette with 60%VG and 40%PG is widely used. Additional file 1: Fig. S1 revealed that lung injury induced by 30% VG solution was equal to lung injury induced by 60%VG solution. The toxicity of 30% VG solution has been confirmed in a previous study using human airway cell lines [21]. Intratracheal instillations of the 20% mannitol (1098 mOsm/L) did not induce lung injury and fibrosis in mice (Additional file 1: Fig. S1).

Intratracheal instillations of 30% VG solution was used to induce VG-induced ALI. In the VG group, mice received a 50 µL 30% VG solution daily on Days 1–4 and received PBS on Day 5. In the VG + LPS group, mice received a 50 µL 30% VG solution daily on Days 1–4 and received LPS on Day 5. In the LPS + p38 inhibitor and VG + LPS + p38 inhibitor groups, each mouse received an intraperitoneal injection of 5 mg/kg p38 inhibitor (SB203580, #tlrl-sb20, InvivoGen, San Diego, CA) 1 h before LPS instillation [22]. In the VG + p38 inhibitor group, each mouse received an intraperitoneal injection of 5 mg/kg p38 inhibitor, 1 h before VG instillation on Day 1. The p38 inhibitor was dissolved in 30 µL dimethyl sulfoxide and mixed with 270 µL normal saline.

Mice were euthanized 24 h after the last intratracheal injection. Lung tissues were collected from each mouse to assess ALI via H&E histology, IHC, IF, and western blot analyses.

### Histological and IHC analyses

Lung tissue was excised 24 h after LPS-induced lung injury, fixed in 4% paraformaldehyde for 10 min, embedded in paraffin, and cut into 4-µm-thick sections. Staining for Ly6G (LS-C36561, 1:100; LifeSpan Biosciences, Seattle, WA), MPO (SC-52707, 1:100, Santa Cruz Biotechnology, Dallas, TX), VLA-4 (#8440S, 1:1000; Cell Signaling, Danvers, MA), VCAM-1 (#14694, 1:1000; Cell Signaling), and TGF-β (ab66043, 1:100, Abcam, Cambridge, UK) was performed using Envision® + Dual Link System-HRP (DAB+) kits (K4065; DAKO, Carpinteria, CA). The sections were deparaffinized in xylene, dehydrated in ethanol, and then heated in 0.01 M citrate buffer (pH 6.0). Endogenous peroxidase activity was inactivated in 3% H_2_O_2_ for 10 min at room temperature (RT), and the sections were blocked with blocking buffer. Secondary anti-rabbit antibody-coated polymer peroxidase complexes were applied for 30 min at RT, followed by substrate/chromogen incubation for 5–15 s at RT. The sections were counterstained with hematoxylin (109249; Merck) for 10 s and then washed in running water for 10 min. They were observed and photographed with an Olympus AX80 fluorescence microscope (Olympus America, Melville, NY). The percentage of IHC signal per photographed field was determined with Image-Pro Plus software (Media Cybernetics, Inc., Silver Spring, MD).

### Lung injury scoring

Two investigators evaluated each H&E-stained slide independently while blind to the groups. To generate the lung injury score as an index of ALI severity, 300 alveoli were counted on each slide at 400× magnification. Within each field, points were assigned according to established criteria [8–13]. We calculated the scores using the following formula: Lung injury score = [(alveolar hemorrhage points/no. of fields) + 2 × (alveolar infiltrate points/no. of fields) + 3 × (fibrin points/no. of fields) + (alveolar septal congestion/no. of fields)]/total number of alveoli counted.

### Masson’s trichrome staining

Lung specimens were fixed in 4% paraformaldehyde for 10 min, embedded in paraffin, and cut into 3-µm-thick sections. The sections were stained with a Trichrome Stain Kit (#ab150686, Abcam, Cambridge, UK) according to the manufacturer’s instructions.

### Ashcroft scale

Two investigators evaluated each Masson’s trichrome-stained slide independently and blind to group assignments. Points were assigned within each field pursuant to the predetermined criteria used in a previous study [23].

### Western blotting

Mouse lung tissues were homogenized in lysis buffer [RIPA lysis buffer (475 uL), Halt protease inhibitor cocktail (5 uL), and 0.1 M Na3VO4 (20 uL); Thermo Fisher Scientific, MA], centrifuged at 20,000 rpm at 4 °C for 10 min, and stored at − 20 °C. Equal amounts of protein homogenate were resolved on 7.5–10% sodium dodecyl sulphate–polyacrylamide electrophoresis gels and transferred to polyvinylidene fluoride membranes. The blots were blocked in Tris-buffered saline with Tween (TBST) containing 5% milk and probed with primary antibodies to VLA-4 (#8440S, 1:1000; Cell Signaling), VCAM-1 (#14694, 1:1000; Cell Signaling), p38 (#9212S, 1:1000; Cell Signaling), phosphorylated (p)-p38 (#9211, 1:1000; Cell Signaling), collagen-1 (ab34710, 1:1000; Abcam), TGF-β (ab66043, 1:1000, Abcam), and β-actin (20536-1-A, 1:5000; Proteintech). The blots were washed in TBST, incubated with horseradish peroxidase-conjugated secondary antibodies (goat anti-rabbit immunoglobulin G; H&L, ab6721; Abcam], and detected using an enhanced chemiluminescence substrate (Pierce Biochemicals). Each blot was exposed to film, and the densitometry of the immunoreactive bands was performed in ImageJ.

### Immunofluorescence

Cells from BALF and blood were subjected to cytospinning, fixed, permeabilized, and stained with Ly6G (LS-C36561, 1:100; LifeSpan Biosciences) or MPO (ab9525, 1:100, abcam) antibodies as primary antibodies. The following day, goat anti-rabbit IgG (H&L) Alexa Fluor® 488 (1:400, ab150077; Abcam) or goat anti-rabbit IgG (H&L) Cy5® (ab6564, 1:400; Abcam) was incubated as a secondary antibody at 37 °C for 2 h. Cell nuclei were counterstained with DAPI (H-1200; Vector Laboratories, CA). Images of the cells were obtained under a Fluoview confocal microscope (FV10i; Olympus).

### Statistical analysis

To limit the variability of each experimental condition, all mice were prepared and studied at the same time. Separate groups of mice were used for lung injury scoring, IHC, flow cytometry, and migration assays. The data are presented as means ± standard errors or means ± standard deviations and were analyzed using a one-way analysis of variance and the Tukey–Kramer multiple comparisons test (for multiple groups) or Student’s t Test (for two groups). P values < 0.05 were considered statistically significant.

## Conclusions

We have identified a novel role of the main e-cigarette component VG in the enhancement of endotoxin-induced ALI via the activation of neutrophil chemotaxis and fibrosis. This effect is mediated by upregulation of adhesion molecule expression and fibrogenesis and p38 MAPK pathway activation by VG. These findings provide important evidence that this e-cigarette component has a specific pro-inflammatory effect in an ALI setting and warrants support in future EVALI management.

## Supplementary Information

**Additional file 1: Figure S1. **Histological examination of VG-induced lung injury. (A) Histological examination of H&E-stained sections revealed that administration of 30% VG or 60% VG induced the histological features of acute lung injury (ALI) in mice. (B-C) In contrast, 20% mannitol did not induce ALI or lung fibrosis in mice. Data sets are expressed as means ± standard deviations. *p < 0.05 vs. PBS; N = 6 per group.

## Data Availability

The datasets used and/or analysed during the current study are available from the corresponding author on reasonable request.
