# Supplementary material for: The main e-cigarette component vegetable glycerin enhances neutrophil migration and fibrosis in endotoxin-induced lung injury via p38 MAPK activation
Source: Respir Res. 2023 Jan 10;24:9. doi: 10.1186/s12931-022-02307-z (PMC9832808; doi:10.1186/s12931-022-02307-z)

**Figure S1.** **Histological examination of VG-induced lung injury.** (A) Histological examination of H&E-stained sections revealed that administration of 30% VG or 60% VG induced the histological features of acute lung injury (ALI) in mice. (B-C) In contrast, 20% mannitol did not induce ALI or lung fibrosis in mice. Data sets are expressed as means ± standard deviations. *p < 0.05 vs. PBS; N = 6 per group.


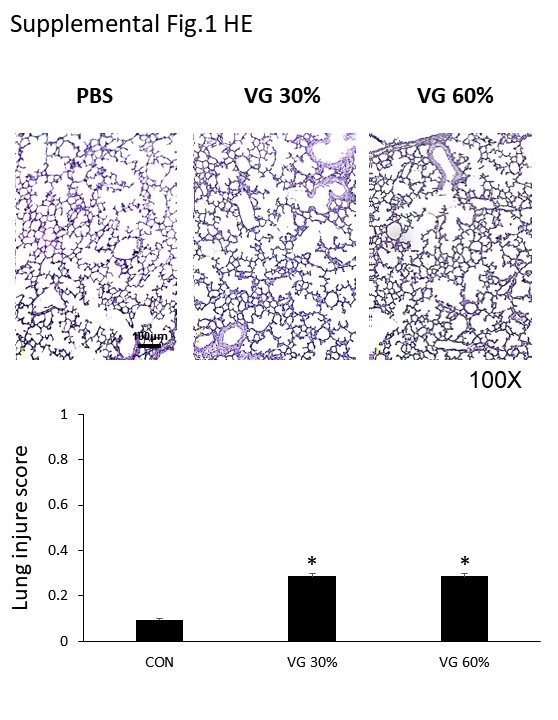


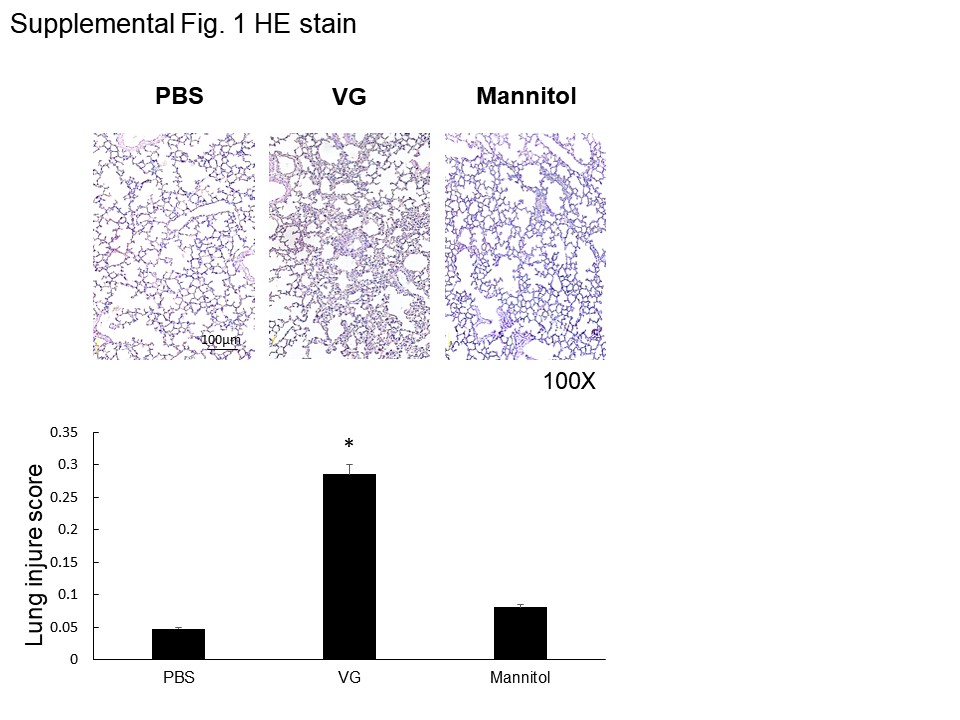


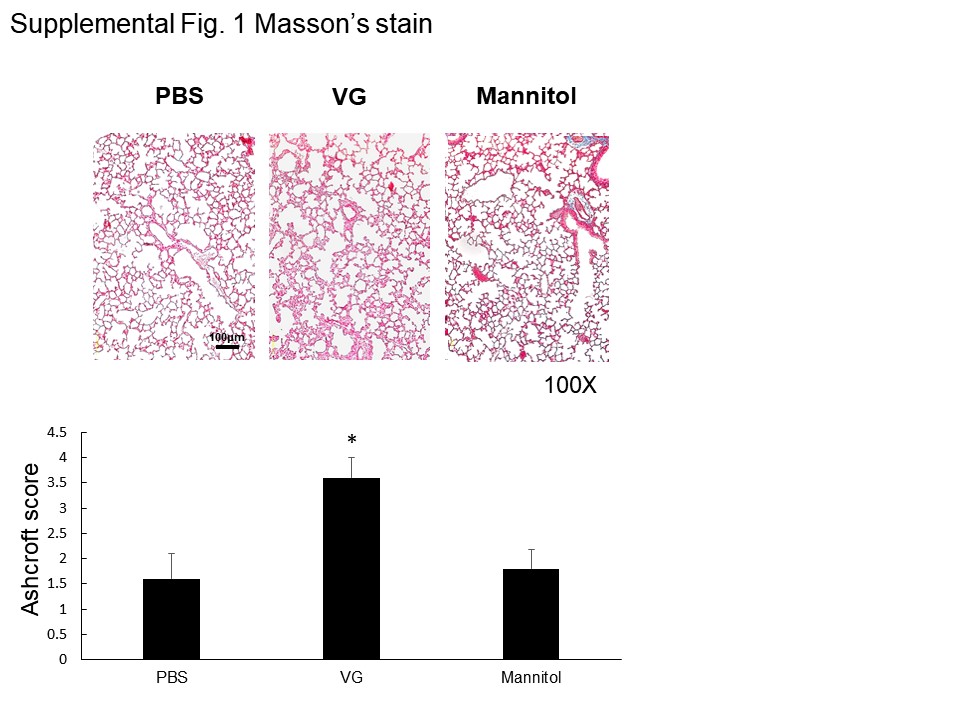

Supplement: Supplementary file 1 — Additional file 1: Figure S1. Histological examination of VG-induced lung injury. (A) Histological examination of H&E-stained sections revealed that administration of 30% VG or 60% VG induced the histological features of acute lung injury (ALI) in mice. (B-C) In contrast, 20% mannitol did not induce ALI or lung fibrosis in mice. Data sets are expressed as means ± standard deviations. *p < 0.05 vs. PBS; N = 6 per group. [file 12931_2022_2307_MOESM1_ESM.docx]
